# Cellular prion protein levels modulate nigrostriatal vulnerability to α-synuclein oligomers in Parkinson’s disease

**DOI:** 10.3389/fnins.2026.1913279

**Published:** 2026-09-17

**Authors:** Flavio de Souza Júnyor, Felipe Saceanu Leser, Sarah de Souza Albuquerque, Rachel de Barros-Telles, Luana Heimfarth, Brenda da Silva Andrade, Carla Moreira Furtado, Lorena Fortuna da Silva, Felipe Cauã Pinheiro dos Santos, Anna Leandra Sant'Anna Ramos de Oliveira, Sergio T. Ferreira, Carolina Braga, Celina Garcia da Fonseca, Gilda Angela Neves, Luciana Romão, Flavia Regina Souza Lima

**Affiliations:** 1 Laboratory of Glial Cell Biology, Biomedical Sciences Institute, Universidade Federal do Rio de Janeiro, Rio de Janeiro, RJ, Brazil; 2 Department of Internal Medicine, Cardiovascular Research Center, Yale University School of Medicine, New Haven, CT, United States; 3 Laboratory of Molecular Pharmacology, Biomedical Sciences Institute, Universidade Federal do Rio de Janeiro, Rio de Janeiro, RJ, Brazil; 4 Institute of Biophysics Carlos Chagas Filho, Universidade Federal do Rio de Janeiro, Rio de Janeiro, RJ, Brazil; 5 Institute of Medical Biochemistry Leopoldo de Meis, Universidade Federal do Rio de Janeiro, Rio de Janeiro, RJ, Brazil; 6 Laboratory of Neurobiology Applied to Biomedicine, Biomedical Sciences Institute, Universidade Federal do Rio de Janeiro, Rio de Janeiro, RJ, Brazil; 7 Núcleo Multidisciplinar de Pesquisas em Biologia, NUMPEX-Bio, Universidade Federal do Rio de Janeiro, Duque de Caxias, RJ, Brazil; 8 Biomedical Sciences Institute, Universidade Federal do Rio de Janeiro, Macaé, RJ, Brazil

**Keywords:** cellular prion protein, neurodegeneration, neuroinflammation, Parkinson’s disease, α-synuclein oligomers

## Abstract

α-Synuclein oligomer (αSynO)-mediated neurodegeneration is a hallmark of Parkinson’s disease (PD) pathogenesis, yet the molecular mechanisms mediating this toxicity remain elusive. In this study, we investigated the cellular prion protein (PrP^C^) as a key modulator of αSynO-induced neurodegeneration using an integrative translational approach combining human transcriptomic analysis with *in vivo* bidirectional genetic modulation in mice. Bioinformatics analysis of postmortem substantia nigra samples revealed that the *PRNP* gene is significantly upregulated in idiopathic PD patients, with its co-expression network enriched for plasma membrane raft organization and synaptic vesicle dynamics. Single-cell RNA-seq confirmed that *PRNP* expression is predominantly neuronal and expressed at low levels in microglia. To validate these findings, we performed intrastriatal αSynO injections in PrP^C^-knockout (PrnpKO), PrP^C^-overexpressing (Tg20), and wild-type (WT) mice. Immunohistochemical analysis at 30 days post-injection revealed that dopaminergic vulnerability was determined by PrP^C^ expression levels. Tg20 mice exhibited reduced tyrosine hydroxylase immunoreactivity in the caudate-putamen, while PrnpKO mice were protected. Behavioral assessment showed that PrP^C^ levels modulate baseline motor performance, with Tg20-αSynO mice consistently displaying the most severe motor impairment. RT-qPCR analysis of striatal transcripts/mRNA levels revealed genotype-specific neuroinflammatory responses to αSynO. WT mice mounted a selective anti-inflammatory *Arginase 1* response, PrnpKO mice partially resolved their basal pro-inflammatory profile, whereas Tg20 mice exhibited pro-inflammatory exacerbation with significant upregulation of *Tnfα* and *Il1β*. Together, these findings identify PrP^C^ expression levels as an important modulator of nigrostriatal vulnerability and neuroinflammatory phenotype in response to αSynO, supporting the hypothesis that PrP^C^ contributes to αSynO-mediated pathogenic mechanisms.

## 1 Introduction

Parkinson’s disease (PD) is recognized as the second most common neurodegenerative disorder after Alzheimer’s disease, affecting approximately 11.8 million individuals worldwide in 2021 (GBD 2021 Nervous System Disorders Collaborators, 2024), with projections estimating this number of cases will exceed 25 million by 2050 (Su et al., 2025). Clinically, PD manifests through a constellation of motor impairments (bradykinesia, resting tremor, and rigidity) frequently accompanied by debilitating non-motor features including hyposmia, autonomic disturbances, sleep disorders, and cognitive decline (Armstrong and Okun, 2020; Tanner and Ostrem, 2024).

Pathologically, the disease is characterized by the loss of dopaminergic neurons predominantly in the substantia nigra pars compacta and the consequent degeneration of their projections to the striatum, the nigrostriatal pathway (Bloem et al., 2021). A hallmark of this process is the accumulation of intracellular inclusions termed Lewy bodies, mainly composed of fibrillar α-synuclein (Morris et al., 2024). However, mounting evidence points to soluble, oligomeric species as the principal toxic drivers of neurodegeneration, disrupting synaptic, mitochondrial, and lysosomal function and propagating pathology between cells (Ono, 2017; Paleologou et al., 2009; Emin et al., 2022). The distribution of α-synuclein pathology extends beyond the substantia nigra to involve multiple regions of the extrapyramidal system, brainstem nuclei, and the peripheral autonomic nervous system (Choong and Mochizuki, 2022).

While the extracellular transfer and cell-to-cell propagation of α-synuclein aggregates involve multiple pathways (Xie et al., 2022; Mavroeidi et al., 2022; Dilsizoglu Senol et al., 2021), the identity of the specific cell-surface receptors that mediate the binding and downstream neurotoxic signaling of these aggregates remains a critical open question. In this context, the cellular prion protein (PrP^C^) has emerged as a potential cell-surface mediator of α-synuclein toxicity. Several reports have demonstrated that PrP^C^ binds preferentially to oligomeric α-synuclein (αSynO), triggering downstream signaling cascades involving Fyn kinase phosphorylation and NMDA receptor activation, which culminate in synaptic impairment and neuronal dysfunction (Grimaldi et al., 2022; Vieira et al., 2024; Thom et al., 2022; Aulić et al., 2017; Ferreira et al., 2017). However, some studies have failed to detect direct binding between PrP^C^ and αSynO or have reported aggregate-induced toxicity in PrP^C^-null models (La Vitola et al., 2019; Bigi et al., 2025). These conflicting findings may stem from differences in experimental conditions, including the conformational state and preparation method of α-synuclein aggregates used, the cellular models and species examined, the binding assays employed, and the specific PrP^C^ domains or epitopes assessed. This duality highlights the complexity of α-synuclein propagation mechanisms and underscores the need for further investigation into the precise molecular interactions that govern disease progression.

To elucidate the relevance of PrP^C^ in PD, we investigated how the bidirectional modulation of PrP^C^ expression alters the neuroinflammatory profile, dopaminergic neuronal loss, and behavioral phenotypes, following intrastriatal stereotaxic injection of αSynO. Utilizing murine models with *PRNP* gene deletion (PrnpKO) and overexpression (Tg20), we determined whether the absence or excess of this cell-surface receptor modulates the severity of α-synuclein-induced neurotoxicity, characteristic of PD.

## 2 Methods

Full details of the methodology and materials are available in Supplementary material 1.

## 3 Results

### 3.1 *PRNP* is upregulated in idiopathic Parkinson’s disease and is associated with neuronal vulnerability pathways

To investigate the translational relevance of PrP^C^ in PD, we performed an integrative bioinformatics analysis combining bulk transcriptomic and scRNA-seq datasets from idiopathic Parkinson’s disease (IPD) patients and healthy controls (GSE7621, GSE157783).

First, we analyzed a microarray dataset derived from postmortem SNpc samples (GSE7621; 16 PD and 9 controls). Differential expression analysis showed that *PRNP* was significantly upregulated in idiopathic PD compared with controls (Figure 1a).

**Figure 1:**
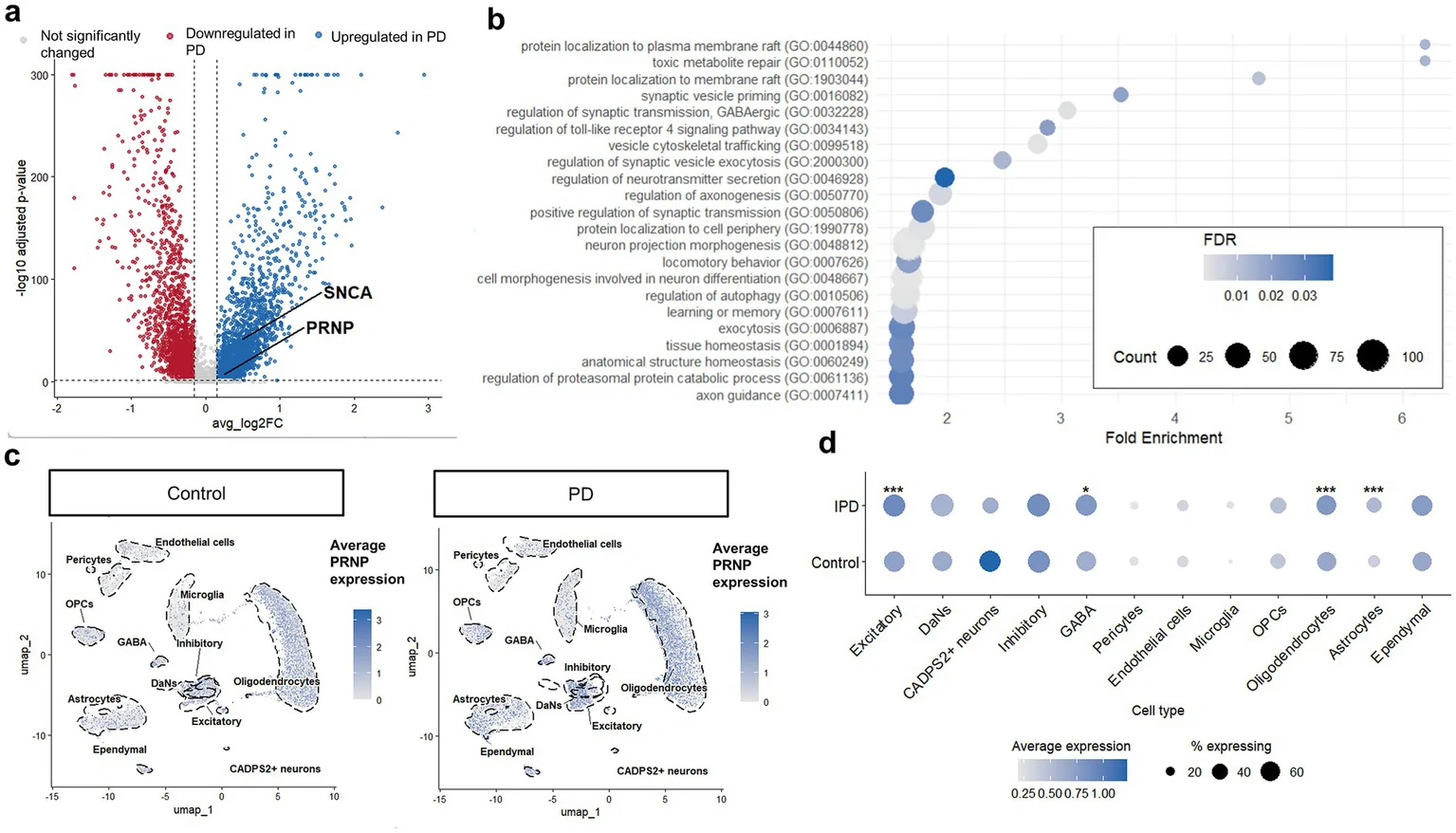
*PRNP* is upregulated in idiopathic Parkinson’s disease and associated with neuronal vulnerability pathways. **(a)** Volcano plot showing differential gene expression analysis of postmortem SNpc microarray samples from individuals with IPD (*n* = 16) and neurologically healthy controls (*n* = 9) (GSE7621). Differentially expressed genes were defined as those with log_2_ fold change (log_2_FC) > 0.15 and adjusted *p* < 0.05 (Benjamini-Hochberg correction). *PRNP* and *SNCA*, encoding PrP^C^ and α-synuclein, respectively, are highlighted. *PRNP* expression was significantly increased in PD samples (log_2_FC = 0.177, 1.13-fold increase; adjusted *p* = 3.15 × 10^−4^). **(b)** GO enrichment analysis of genes significantly correlated with *PRNP* expression in the IPD group from GSE7621 (Spearman’s |*r*| > 0.15 and *p* < 0.05). The dot plot displays enriched GO biological process terms, fold enrichment, false discovery rate (FDR), and the number of genes associated with each term. GO enrichment was performed using the PANTHER Overrepresentation Test (release 20,240,807) with Fisher’s exact test and Benjamini-Hochberg FDR correction. Only terms with FDR < 0.05 are shown. The reference list consisted of all annotated *Homo sapiens* genes in the PANTHER database (20,580 genes). **(c,d)** Cell type-resolved *PRNP* expression analysis using single-nucleus RNA sequencing data from human midbrain samples (GSE157783; 6 controls and 5 IPD cases). **(c)** UMAP projections showing annotated cell populations. **(d)** Dot plot showing average *PRNP* expression (color intensity) and the percentage of *PRNP*-expressing cells (dot size) across cell types in control and IPD samples. Statistical significance was assessed using a two-sided Wilcoxon rank-sum test comparing individual cells from IPD and control groups within each cell type. *p* values were adjusted for multiple testing using the Benjamini–Hochberg false discovery rate (FDR) test. FDR < 0.05*, FDR < 0.01**, and FDR < 0.001***.

To further explore the molecular context of *PRNP* in PD, we performed a gene co-expression analysis within the IPD group by correlating *PRNP* expression with all detected transcripts. Genes significantly correlated with *PRNP* were subjected to GO enrichment analysis. This approach identified protein localization to plasma membrane rafts as the most enriched biological process (fold enrichment > 6). In addition, the *PRNP*-associated gene network was enriched for pathways associated with neuronal homeostasis, including synaptic vesicle priming, vesicle-mediated transport, cytoskeletal trafficking, and regulation of autophagy (Figure 1b).

To compare changes in the *PRNP*-associated transcriptional signature between PD patients and non-neurological individuals, the same correlation analysis was performed in the control samples. We identified 3,201 *PRNP*-correlated genes in PD and 3,120 in controls, of which 576 were shared between the two groups (Supplementary Figure 1a). Among these shared genes, 309 were negatively correlated with *PRNP* in both groups and 122 were positively correlated in both groups, whereas 137 showed correlations in opposite directions. Thus, approximately 75% of the shared *PRNP*-associated genes showed concordant directions of association between PD and controls (Supplementary Figure 1b).

At the transcriptome-wide level, *PRNP*-gene correlation coefficients showed only weak overall concordance between PD and controls (Spearman *ρ* = 0.167). When restricted to the *PRNP*-gene correlation coefficients *PRNP*-associated network showed moderate overall concordance between PD and controls (Spearman *ρ* = 0.374), suggesting that a subset of the *PRNP*-associated transcriptional network is preserved between the two groups, while many associations differ in magnitude or direction.

To functionally characterize changes in the *PRNP*-associated gene expression correlation network between PD and controls, we performed Gene Set Enrichment Analysis (GSEA) using the difference in *PRNP*-gene correlation coefficients between groups (Δ*ρ* = *ρ*_PD − *ρ*_Control) as the ranking metric. This analysis revealed a clear functional organization of the correlation shifts. Genes involved in neurotransmission and synaptic function (*SNAP25, STXBP1, SYT1*, and *RIMS1*) were significantly enriched toward positive Δ*ρ* values, indicating a systematic shift toward more positive *PRNP*-gene correlations in PD (Supplementary Figures 1c,d). Conversely, genes involved in immune responses, antigen processing, and interferon signaling, neuronal survival, including NF-κB signaling and neuronal apoptotic processes (*RELA, TNFAIP3, SQSTM1, BDNF, BCL2, NR4A2*) (Supplementary Figures 1c,e), as well as protein homeostasis responses to misfolded/unfolded proteins (*UBE2K, UCHL5, USP7*) (Supplementary Figures 1c,f) were significantly enriched toward negative Δ*ρ* values, indicating a shift toward more negative *PRNP*–gene correlations in PD. Together, these findings suggest that the *PRNP*-associated transcriptional landscape differs between PD and controls along two major functional axes involving synaptic/neurotransmission pathways and immune/proteostasis-related processes. These analyses indicate that the *PRNP*-associated transcriptional network is only partially conserved between PD and controls, with coordinated disease-associated shifts particularly involving synaptic/neurotransmission, immune, and proteostasis-related processes.

To define the cellular sources of *PRNP* expression at the level of central nervous system (CNS) cell subtypes, we next analyzed single-nucleus RNA-seq data from human midbrain samples (GSE157783; 6 controls and 5 IPD cases). Cell-type-resolved analysis revealed that *PRNP* expression is predominantly neuronal, with strong enrichment in excitatory, inhibitory, and GABAergic neurons, as well as detectable expression in astrocytes and ependymal cells. Notably, *PRNP* was also highly expressed in oligodendrocytes. In contrast, microglial expression of *PRNP* was minimal, indicating a limited direct contribution of immune cells to its transcriptomic profile under both physiological and disease conditions (Figure 1c).

Importantly, when comparing IPD and control samples, *PRNP* expression exhibited cell-type-specific differences. Compared to controls, increased expression was observed in excitatory neurons, inhibitory neurons, GABAergic neurons, and oligodendrocytes in IPD. In contrast, dopaminergic neurons (DaNs) showed a slight reduction in *PRNP* expression in IPD (Figure 1d).

Overall, our integrative analysis shows that *PRNP* is significantly upregulated in idiopathic PD and is associated with gene networks related to synaptic function, membrane microdomain organization, immune responses, and proteostasis regulation. At the cellular level, *PRNP* expression is predominantly neuronal and displays cell-type-specific modulation in PD, with increased levels in excitatory and inhibitory neurons.

### 3.2 PrP^C^ expression levels modulate baseline motor phenotypes and αSynO-induced dopaminergic vulnerability

To investigate the specific role of PrP^C^ in the pathogenesis of PD, we employed an αSynO-induced mouse PD model. To assess how the bidirectional modulation of PrP^C^ expression alters the susceptibility to PD-like features, we stereotaxically injected 2 μg αSynO into the *caudate-putamen* (CPu) of PrP^C^-knockout mice (PrnpKO), as well as in mice that overexpress this protein (Tg20) and their respective controls (WT; 129Sv × C57BL/6 and C57BL/6 mice, respectively). All animals underwent baseline motor-performance evaluations prior to surgery, followed by a comprehensive battery of five distinct motor and non-motor behavioral assessments at 28–30 days post-injection (dpi) (Figure 2A). The 30-day post-injection endpoint was selected based on our previous study (Leser et al., 2025), which identified this time point as optimal for detecting behavioral deficits, neuroinflammation, and dopaminergic neuronal loss following intrastriatal α-synuclein injection. It provides sufficient time to investigate acute toxicity mechanisms and pathological changes induced by α-SynO while minimizing the confounding effects of acute surgical inflammation observed at earlier time points and the potential compensatory mechanisms or ceiling effects associated with later stages.

To determine whether PrP^C^ expression influences susceptibility to αSynO-induced dopaminergic pathology, TH immunoreactivity was quantified in the ipsilateral caudate-putamen 30 days after injection (Figures 2b,c). WT mice showed reduced TH immunoreactivity following αSynO administration. This reduction was absent in PrnpKO mice, whereas Tg20 mice exhibited a loss of TH immunoreactivity relative to their respective vehicle-treated controls.

**Figure 2:**
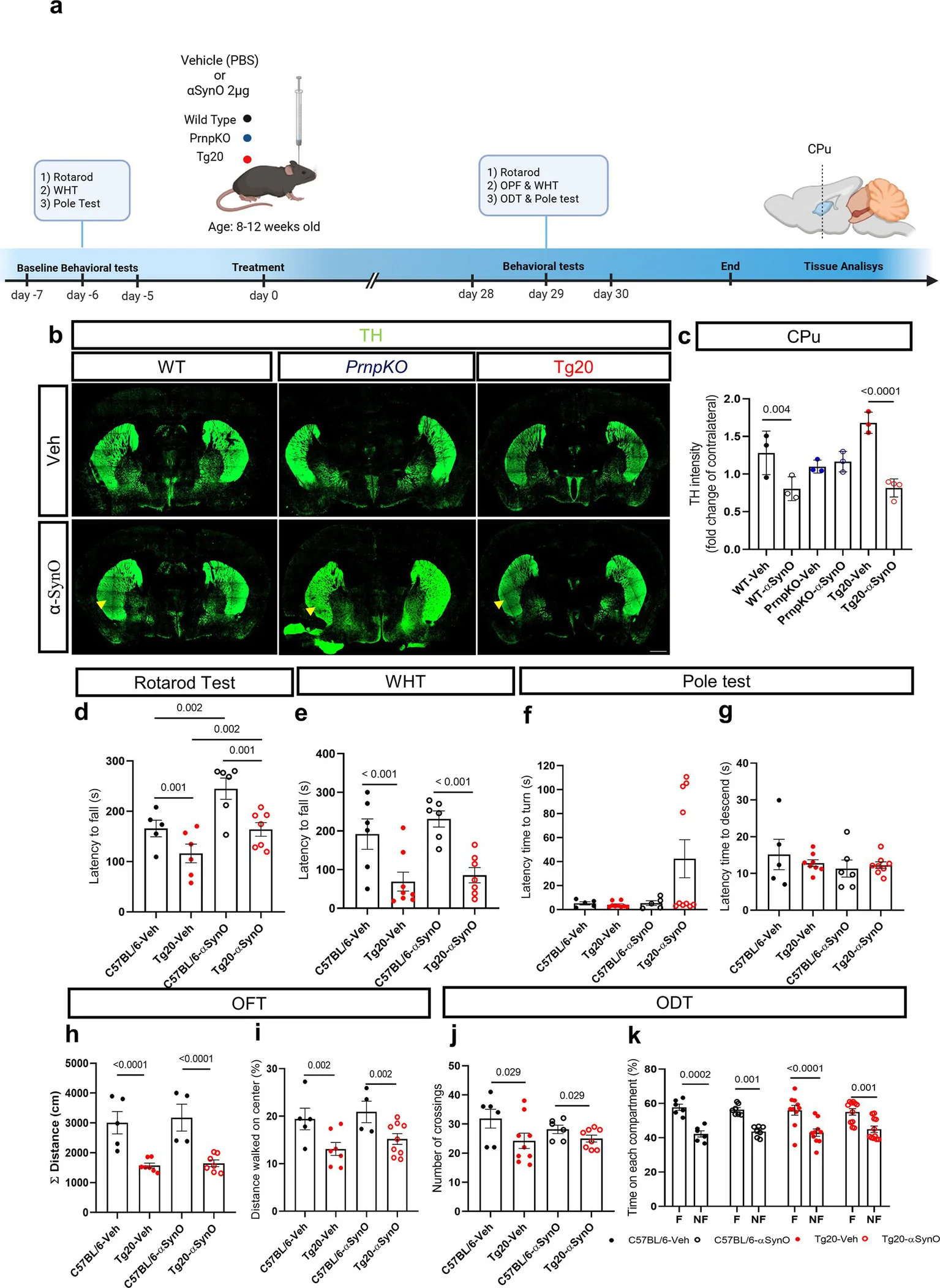
PrP^C^ expression modulates striatal dopaminergic denervation and motor behavior in the αSynO mouse model. **(a)** Schematic representation of the experimental design. Mice underwent baseline motor testing 7 days before surgery. On day 0, mice received intrastriatal injections of vehicle (Veh) or 2 μg αSynO. Behavioral testing was performed at 28–30 dpi, followed by brain collection for histological analysis. **(b)** Representative TH immunostaining in the CPu of WT (129/Sv × C57BL/6), PrnpKO, and Tg20. White arrows indicate the injection side. Scale bar = 200 μm. **(c)** Quantification of TH immunoreactivity in the ipsilateral CPu of WT (129/Sv × C57BL/6), PrnpKO, and Tg20, normalized to the contralateral side. **(d–g)** Motor test performance at 28–30 dpi: Latency to fall in the rotarod **(d)**, latency to fall in the wire hanging test (WHT; **e**), latency to turn downward in the pole test **(f)**, and latency to descend the pole **(g)**. **(h,i)** Open field test assessment at 29 dpi, showing total distance traveled **(h)** and distance traveled in the center zone **(i)**. **(j,k)** Olfactory discrimination test at 30 dpi, showing the number of crossings between compartments **(j)** and time spent investigating familiar (F) and non-familiar (NF) odors **(k)**. Statistical analysis was performed using two-way ANOVA followed by Tukey’s multiple-comparisons test. Data are presented as mean ± SEM. (*n* = 4–9/group).

Having demonstrated a PrP^C^ levels effect on dopaminergic structural integrity, we next assessed whether this vulnerability translated into detectable behavioral deficits. To evaluate motor deficits following αSynO injection, we assessed gross motor coordination and neuromuscular strength using the rotarod and WHT (wire hanging test), respectively. In the rotarod, a clear genotype effect was observed. In both vehicle and αSynO-injected groups, Tg20 mice displayed a reduced latency to fall relative to C57BL/6 mice (Figure 2d). Moreover, an injection effect was also observed, with αSynO-injected groups showing a longer fall latency regardless of the strain. In the WHT, Tg20-Veh mice already exhibited a reduced latency to fall compared to C57BL/6-Veh controls, revealing an intrinsic neuromuscular deficit associated with PrP^C^ overexpression. This difference persisted following the αSynO treatment, with Tg20-αSynO mice performing worse than C57BL/6-αSynO mice (Figure 2e).

The fine motor coordination was assessed using the pole test. No significant differences in turning latency were detected between Tg20-αSynO and Tg20-Veh mice. No significant differences in turning latency were observed between C57BL/6-Veh and C57BL/6-αSynO mice, nor between Tg20-αSynO and C57BL/6-αSynO mice (Figure 2f). No significant differences were detected in total descent time across any groups (Figure 2g).

In the OFT, Tg20 mice exhibited reduced total distance traveled compared with C57BL/6, under both vehicle and αSynO conditions (Figure 2h). Also, a clear strain effect was observed in the field center exploration, where Tg20 mice traveled a shorter distance in the more aversive area of the OF regardless of treatment (Figure 2i). In the ODT, all groups successfully discriminated between familiar and novel odors, with no significant differences in discrimination index across genotypes or treatment conditions (Figure 2k). The number of crossings between compartments was also affected by genotype (Figure 2j), confirming the basal locomotor impairment of Tg20 mice observed in the OFT.

For PrnpKO mice, no significant differences were observed in the rotarod or WHT between genotypes or treatment conditions (Supplementary Figures 2a,b). In the pole test, however, a significant effect of genotype was detected, with PrnpKO mice exhibiting increased latency to turn and total descent time compared to 129Sv × C57BL/6 controls, both under vehicle (PrnpKO-Veh vs. 129Sv × C57BL/6-Veh) and αSynO conditions (PrnpKO-αSynO vs. 129/Sv-αSynO). Yet, αSynO treatment did not significantly alter pole test performance within either genotype (Supplementary Figures 2c,d).

In the OFT, total distance traveled was comparable between PrnpKO and 129Sv × C57BL/6 mice across all conditions (Supplementary Figure 2e). In contrast, analysis of center distance revealed that PrnpKO-Veh mice traveled significantly more in the center zone compared to 129Sv × C57BL/6-Veh controls, suggesting a reduced anxiety-like phenotype at baseline. Notably, αSynO treatment reversed this pattern, with PrnpKO-αSynO mice displaying significantly reduced center distance compared to PrnpKO-Veh animals (Supplementary Figure 2f).

In the ODT, olfactory discrimination was preserved in PrnpKO mice, with all groups successfully distinguishing between familiar and novel odors regardless of genotype or treatment. No significant difference between groups was observed (Supplementary Figure 2h). However, PrnpKO mice displayed a significantly higher number of compartment crossings than 129Sv × C57BL/6 controls, irrespective of αSynO treatment (Supplementary Figure 2g).

To confirm the efficacy of the αSynO model and assess the influence of genetic background on behavioral susceptibility, we performed the same intrastriatal injection protocol in Swiss outbred mice (Supplementary Figure 3a). In contrast to the C57BL/6 and 129Sv × C57BL/6, Swiss-αSynO mice exhibited a significantly reduced latency to fall in the rotarod (Supplementary Figure 3b) and WHT (Supplementary Figure 3c) compared to Swiss-Veh controls, with a trend toward increased latency to turn in the pole test (Supplementary Figure 3d). No significant difference was detected in total descent time (Supplementary Figure 3e).

### 3.3 PrP^C^ expression dictates the basal inflammatory transcriptional profile and shapes the neuroinflammatory response to αSynO

Effects of surgical inflammation typically resolve within 7–14 days after stereotaxic surgery (Lima et al., 2016). In accordance with our previous findings (Leser et al., 2025), microglial activation and inflammatory markers in vehicle-treated animals differed significantly than αSynO-treated by 30 days. This methodological detail allows the specific assessment of α-synuclein oligomer-induced pathology without residual surgical confounding.

To investigate the impact of PrP^C^ on neuroinflammation, we assessed the transcriptional profile of pro-inflammatory and anti-inflammatory cytokines and mediators in the CPu using RT-qPCR (Figure 3a). Basal analysis (vehicle-treated animals) revealed that the striatal inflammatory transcriptional landscape is intrinsically modulated by PrP^C^ levels. Our data showed that PrP^C^ ablation (PrnpKO mice) induces a basal transcriptional profile enriched in pro-inflammatory markers. Specifically, PrnpKO animals displayed higher levels of pro-inflammatory mediators (*Tnfα*, *Il1b*, and *Mmp9*) when compared to PrP^C^-overexpressing mice (Tg20) (Figures 3b–d). In addition, basal *Mmp9* expression was significantly higher in PrnpKO relative to WT animals. In contrast to the alterations in pro-inflammatory markers, the basal expression of genes associated with a resolutive and anti-inflammatory phenotype (*Arg1*, *Tgfb*, and *Il10*) remained unchanged across the different genotypes (Figures 3a–e).

**Figure 3:**
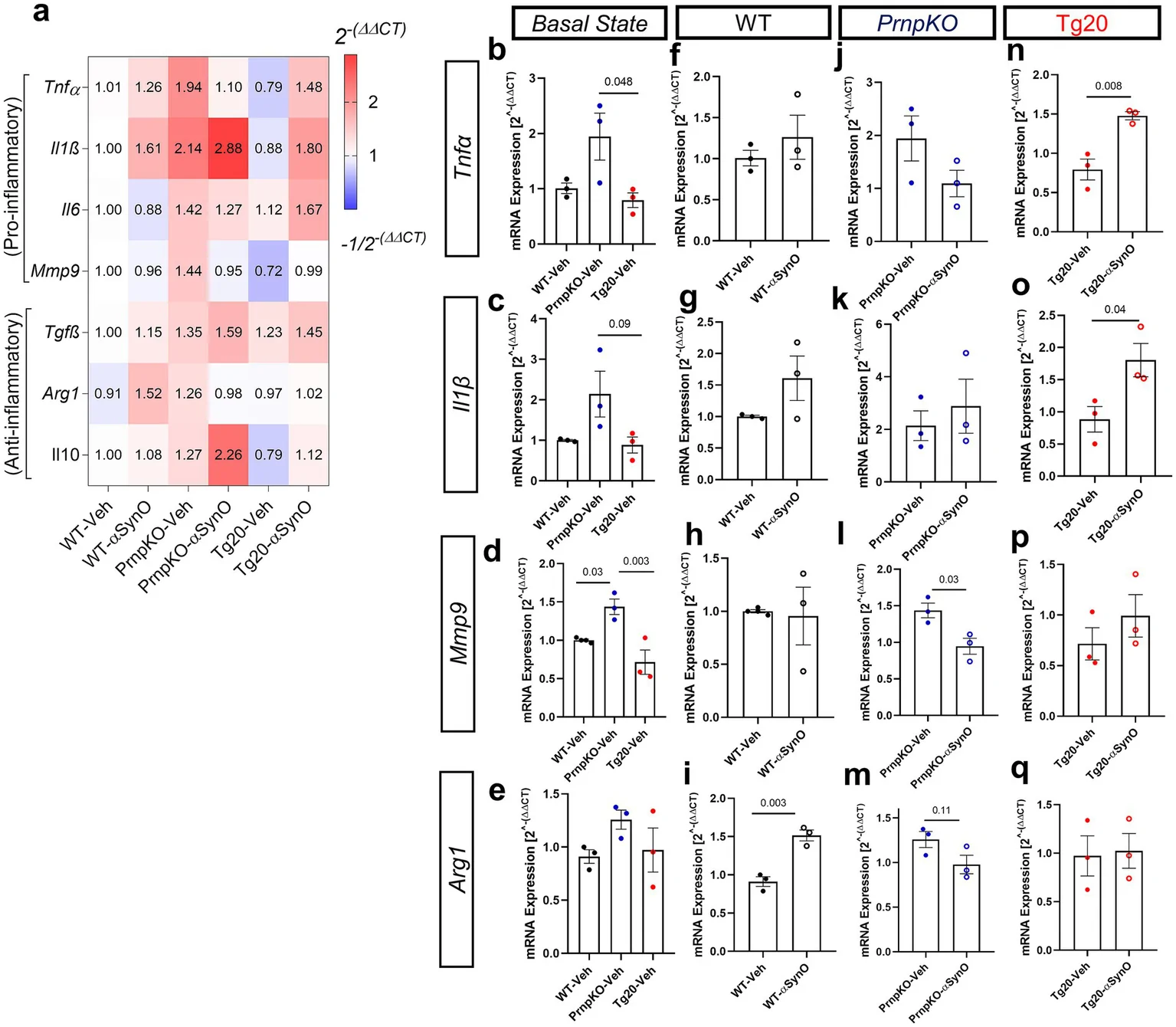
PrP^C^ expression levels differentially modulate basal and αSynO-induced inflammatory transcriptional responses in the CPu. **(a)** Heat map showing relative mRNA expression of pro-inflammatory (*Tnfα, Il1b, Mmp9*) and anti-inflammatory (*Arg1, Tgfb, Il10*) markers in the CPu across different PrP^C^ expression levels (PrnpKO, WT/129/Sv × C57BL/6, Tg20) following vehicle or αSynO treatment. Color intensity represents relative mRNA expression. **(b–e)** Quantification of transcript levels in vehicle-treated animals (WT/129/Sv × C57BL/6, PrnpKO, and Tg20) for **(B)** *Tnfα*, **(c)** *Il1b*, **(d)** *Mmp9*, and **(e)** *Arg1*. Statistical comparison between groups: One-way ANOVA test with Tukey’s multiple comparisons test (*n* = 3/group). **(f–i)** Transcript levels in vehicle-treated *versus* αSynO-treated animals (WT/129/Sv × C57BL/6) for **(f)** *Tnfα*, **(g)** *Il1b*, **(h)** *Mmp9*, and **(i)** *Arg1*. **(j–m)** Transcript levels in vehicle-treated *versus* αSynO-treated animals (PrnpKO) for **(j)** *Tnfα*, **(k)** *Il1b*, **(l)** *Mmp9*, and **(m)** *Arg1*. **(n–q)** Transcript levels in vehicle-treated *versus* αSynO-treated animals (Tg20) for **(n)** *Tnfα*, **(o)** *Il1b*, **(p)** *Mmp9*, and **(q)** *Arg1*. Statistical comparison between groups: Unpaired Student’s *t*-test (two-tailed) (*n* = 3/group). Data are presented as mean ± SEM.

Furthermore, we evaluated the neuroinflammatory response induced by the intrastriatal injection of αSynO by comparing cytokine expression between αSynO-exposed animals and their respective vehicle controls within each genotype. In WT animals, αSynO exposure promoted a significant and selective increase in the anti-inflammatory transcript *Arg1* compared to its control, with no significant changes in *Tnfα*, *Il1β*, or *Mmp9* (Figures 3f–i).

In PrnpKO, which basally exhibited an activated phenotype characterized by elevated *Tnfα, Il1b,* and *Mmp9* expression, αSynO exposure led to a significant reduction in *Mmp9* transcription and a non-significant trend toward reduced *Arg1* expression compared to the PrnpKO-veh group (Figures 3j–m). In contrast, PrP^C^ overexpression altered the course of the pathological response. In Tg20, αSynO exposure markedly exacerbated the inflammatory profile, resulting in a significant increase in the expression of the pro-inflammatory transcripts *Tnfα* and *Il1b* compared to the Tg20-veh group (Figures 3n–q).

### 3.4 PrP^C^ levels regulate baseline and αSynO-induced microglial morphological reactivity in the CPu

To investigate whether PrP^C^-dependent modulation of neuroinflammatory gene expression was accompanied by morphological changes in microglia, the primary cytokine-secreting cells in the context of PD (Leser et al., 2025), we performed a comprehensive quantitative analysis of Iba1-positive cells in the CPu at 30 dpi (Figure 4a).

**Figure 4:**
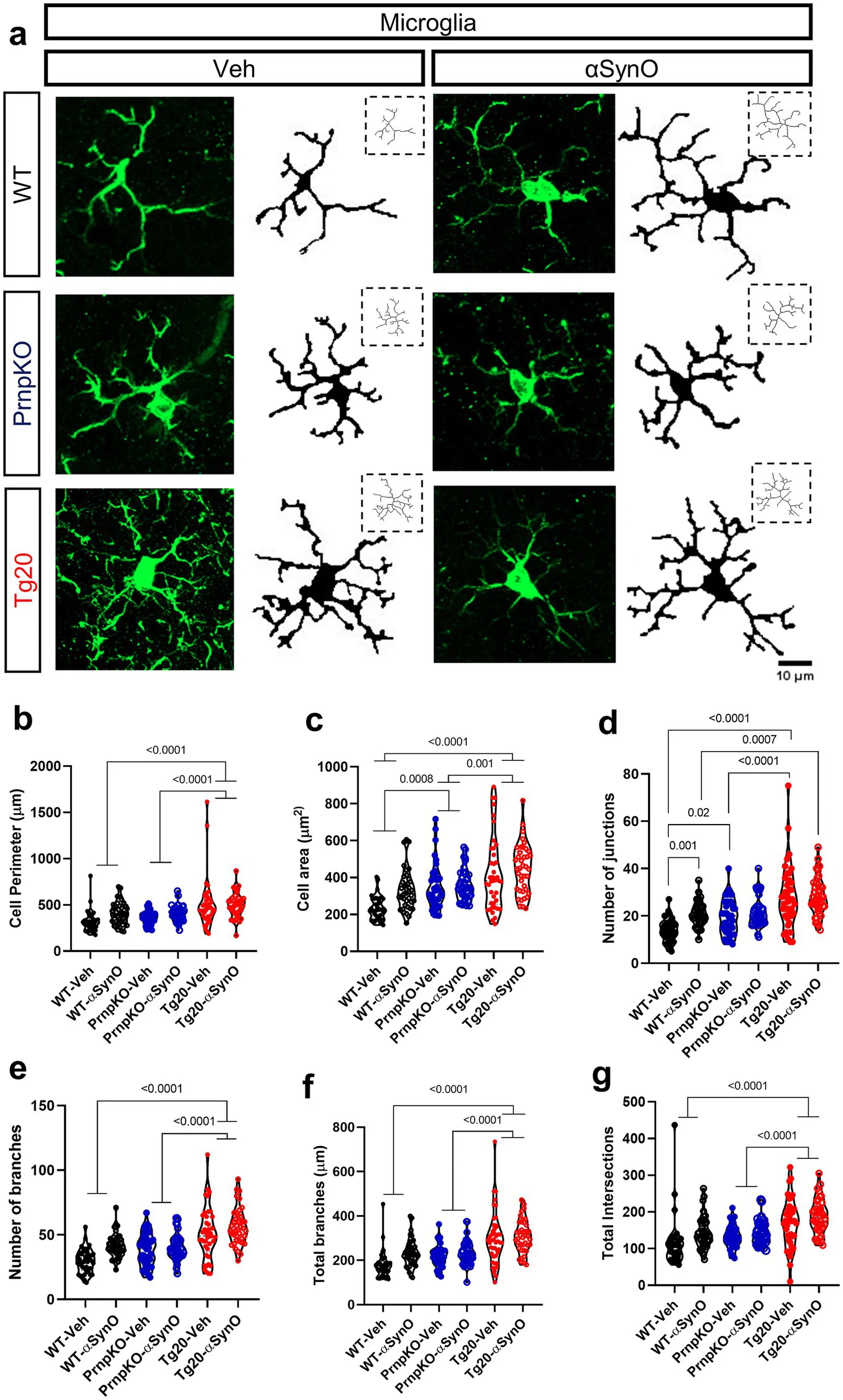
Bidirectional modulation of PrP^C^ alters basal microglial morphology and shapes structural responses to α-synuclein oligomers. **(a)** Representative Iba1 immunostaining in the CPu with image depicting the cellular silhouette and skeletonized structure of Iba1-positive microglial cells in the CPu of mice (WT/129/Sv × C57BL/6, PrnpKO, and Tg20) treated with vehicle or αSynO. Scale bar = 10 μm. **(b–f)** Quantification of morphometric variables of CPu microglial cells of mice treated with vehicle or αSynO. Assessment of cell perimeter **(b)**; *p*_veh x αSynO_ = 0.023, cell area **(c)**; *p*_veh x αSynO_ = 0.001, number of junctions **(d)**, number of branches **(e)**; *p*veh x αSy_nO_ < 0.0001, branch length **(f)**; *p*_veh x αSynO_ = 0.004. Sholl’s analysis of microglial cells from the CPu of mice treated with vehicle or αSynO **(g)**; *p*_veh x αSynO_ = 0.001. Intersection profile of the skeletonized microglial branches with the concentric circles at a given distance from the soma (1 μm step size). Data shown as truncated violin plot. Two-way ANOVA with Tukey’s multiple comparisons test (*n* = 40 cells/group from 3 independent experiments). All data are presented as mean ± SEM.

Under baseline conditions, Tg20 microglia exhibited larger cell perimeters than WT and PrnpKO. Following αSynO treatment, microglia mounted a robust morphological response, with an overall increase in cell perimeter across groups. As a result of these additive effects, Tg20-αSynO microglia maintained elevated perimeters, remaining larger than WT-αSynO (Figure 4b). Basally, both PrnpKO and Tg20 microglia displayed significantly greater cell area compared to WT. Following αSynO exposure, microglia exhibited an overall increase in cell area across all groups. As a result of these additive effects, Tg20-αSynO area remained larger than WT-αSynO (Figure 4c).

At baseline, both PrnpKO-Veh and Tg20-Veh displayed higher numbers of microglial junctions compared to WT-Veh, with Tg20-Veh also exceeding PrnpKO-Veh. Microglial network complexity increased in WT-αSynO. Tg20-αSynO microglia maintained elevated complexity compared to WT-αSynO (Figure 4d). Basally, Tg20 microglia exhibited the highest number of branches among all baseline groups. Tg20-αSynO continued to exhibit increased microglial branch numbers when compared to WT-αSynO (Figure 4e).

Consistent with the other morphometric parameters, Tg20 microglia exhibited greater total microglial arborization length than WT and PrnpKO microglia. Consequently, αSynO exposure induced an overall increase in total arborization length across all groups. There was a trend for Tg20-αSynO to maintain greater total length than WT-αSynO (Figure 4f). At baseline, Tg20 microglia elevated the number of microglial intersections compared to WT and PrnpKO. Following αSynO exposure, microglia exhibited an overall trend toward increased intersections across all groups. Driven by these additive effects, Tg20-αSynO microglia maintained a higher number of intersections compared to WT-αSynO (Figure 4g).

Collectively, these morphological findings provide structural correlates to the transcriptional inflammatory profiles and demonstrate that PrP^C^ levels regulate both the baseline morphological state and the reactive capacity of striatal microglia in response to αSynO.

## 4 Discussion

The present study provides converging human transcriptomic and *in vivo* experimental evidence suggesting the involvement of PrP^C^ in nigrostriatal vulnerability to αSynO-induced neurodegeneration. By integrating differential gene expression analysis in idiopathic PD patients with bidirectional genetic modulation of PrP levels in mice, we demonstrate that PrP^C^ is associated with dopaminergic vulnerability and the secondary neuroinflammatory response.

Our transcriptomic analysis revealed that *PRNP* is significantly upregulated in idiopathic PD, with GO enrichment highlighting plasma membrane rafts and synaptic vesicle dynamics as associated pathways. These findings are consistent with the role of PrP^C^ as a GPI-anchored scaffold protein involved in synaptic signaling and membrane organization (Vieira et al., 2024; Panes et al., 2021). The predominantly neuronal expression pattern establishes a cell-type-specific framework for interpreting the downstream effects observed in our mouse model. Notably, this neuron-predominant upregulation was not uniform across neuronal subtypes. Dopaminergic neurons, the population most vulnerable in PD, showed a slight decrease rather than an increase in *PRNP,* with the net tissue-level upregulation driven mainly by more resistant excitatory/inhibitory neurons and oligodendrocytes. This selective reduction in dopaminergic neurons likely reflects survivorship bias in postmortem tissue, consistent with our *in vivo* data showing that higher PrP^C^ dosage confers greater vulnerability to αSynO-induced dopaminergic loss. This finding regarding neuronal enrichment aligns with Corbett et al. (2020), who identified PrP^C^ as a potential cell-surface receptor for soluble αSyn, tau, and Aβ aggregates in human iPSC-derived neurons, where *PRNP* ablation abolished aggregate-mediated toxicity. The constitutive expression of *PRNP* on neuronal membranes further supports its persistent availability as a cell-surface interactor during disease progression (Vieira et al., 2024). Although the cohort was matched for key variables, the absence of covariate adjustment represents a limitation in interpreting these findings.

*PRNP* was also upregulated in oligodendrocytes in IPD, consistent with growing evidence of oligodendroglial involvement in PD, including α-synuclein-positive inclusions in oligodendrocytes, disrupted myelin-related gene expression, and reduced oligodendrocyte numbers with myelin abnormalities (Bae et al., 2023; Barba-Reyes et al., 2025; Iacono et al., 2026). Given PrP^C^’s known role in oligodendrocyte precursor proliferation and differentiation (Bribián et al., 2012), this upregulation may reflect a reactive or stress-related oligodendroglial phenotype rather than a purely neuronal phenomenon, warranting further investigation into its relationship with changes in PD.

Our findings extend previous evidence implicating PrP^C^ in α-synuclein toxicity by showing that changes in PrP^C^ dosage markedly influence the extent of dopaminergic terminal loss after αSynO exposure. Tg20 mice exhibited marked TH depletion in the CPu following αSynO challenge, whereas PrnpKO mice were protected. However the reduced TH staining may indicate either true terminal degeneration or a phenotypic downregulation of the enzyme due to cellular stress. Future stereological quantifications of dopaminergic neurons in the substantia nigra will shed further light on our current findings. Nevertheless our data are in agreement with previous mechanistic studies showing that PrP^C^ mediates αSyn internalization through clathrin-dependent endocytosis and that PrnpKO mice are protected from oligomeric αSyn accumulation and behavioral deficits in transgenic α-synucleinopathy models (ThySyn and TgM83) (Thom et al., 2022). Similarly, Brás et al. (2018) proposed that PrP^C^-αSynO interaction activates Fyn kinase and NMDA receptor signaling, promoting synaptic dysfunction. Our histological data are consistent with this proposed mechanism, showing that the magnitude of striatal dopaminergic denervation, reflected by decreased TH immunoreactivity in the CPu, scales with PrP^C^ dosage.

The involvement of PrP^C^ in dopaminergic circuitry is further supported by Rial et al. (2014), who demonstrated that PrP^C^ co-localizes with dopaminergic neurons and that its deletion reduces D1 receptor and DARPP-32 expression in the striatum. These intrinsic alterations likely contribute to the baseline behavioral phenotypes observed in our transgenic lines independently of αSynO challenge. Nevertheless, the role of PrP^C^ as an obligatory receptor for αSynO toxicity remains debated. La Vitola et al. (2019) reported that PrnpKO mice developed hippocampal deficits comparable to WT mice following intracerebroventricular αSynO administration. Differences in aggregate preparation, injection route, and brain regions analyzed may explain these discrepancies. Unlike intracerebroventricular delivery, our intrastriatal injections directly target the nigrostriatal pathway, where PrP^C^ is abundantly expressed and functionally relevant (Rial et al., 2014; Salès et al., 1998). However, our model has inherent limitations. Unlike preformed fibril (PFF) models that recapitulate interneuronal propagation and Lewy body-like inclusions, soluble oligomers induce acute neurotoxicity without fully reproducing chronic PD progression (Froula et al., 2019; Cascella et al., 2022). Nevertheless, soluble oligomers are considered the most neurotoxic α-synuclein species, preceding fibril formation and driving early axonal dysfunction, synaptic degeneration, and neuroinflammation (Cascella et al., 2022; Emin et al., 2022; Ingelsson, 2016).

Recently, Bigi et al. (2025) demonstrated that PrP^C^ facilitates the initial interaction of α-synuclein aggregates with neuronal membranes and contributes to early Ca^2+^ influx, whereas sustained toxicity appears to be largely driven by extracellular Ca^2+^-dependent mechanisms. Our data suggest that PrP^C^ overexpression enhances the vulnerability of the nigrostriatal pathway to αSynO, raising the possibility that PrP^C^ may facilitate the early engagement of toxic aggregates with neuronal membranes, as suggested by Bigi et al. (2025).

Our behavioral analyses revealed that PrP^C^ modulation strongly influences baseline motor performance, with Tg20 mice displaying impaired performance in the rotarod, wire hanging, and open field tests, whereas PrnpKO mice exhibited an anxiety phenotype, consistent with previous studies linking PrP^C^ to locomotion and anxiety-related responses (Lobão-Soares et al., 2007; Nico et al., 2005). The relatively limited behavioral effect of αSynO in our work possibly reflects a dominant genotype effect on mice’s behavior. Although αSynO-induced behavioral effects were modest at 30 dpi, Tg20-αSynO mice exhibited the most severe impairments, consistent with the marked TH depletion observed histologically. Importantly, αSynO-injected Swiss mice reproduced the expected nigrostriatal neurodegenerative phenotype, confirming that the αSynO preparations and stereotaxic injection protocol were adequate and effective in inducing a Parkinson’s-like phenotype, independently of the genetic background of the transgenic models used in the main experiments. The relatively limited statistical effect of αSynO possibly reflects a dominant genotype effect on mice behavior. Alternatively, the relatively short post-injection interval may have been insufficient for the full manifestation of αSynO-induced behavioral deficit. Additionally, motor deficits in Parkinson’s disease typically emerge only after approximately 50–70% dopamine depletion, and early-stage motor dysfunction correlates more strongly with striatal terminal loss than with nigral cell body degeneration (Yoo et al., 2026; Rodriguez-Oroz et al., 2009). Further longitudinal studies with extended follow-up periods will be essential to address these questions and determine whether the observed neuropathological alterations ultimately translate into more pronounced functional impairments.

An interesting finding emerging from this study is that PrP^C^ dosage influenced both basal inflammatory transcriptional signatures and the response to αSynO. PrnpKO mice exhibited a basal pro-inflammatory transcriptional signature characterized by elevated *Tnfα*, *Il1b*, and *Mmp9* expression, suggesting that PrP^C^ normally exerts anti-inflammatory effects, consistent with previous reports showing that PrP^C^ attenuates innate immune activation and regulates microglial polarization (Shi et al., 2013; Mantuano et al., 2022). Importantly, this basal inflammatory transcriptional state may contribute to the neuroprotection observed in PrnpKO mice following αSynO challenge. Conversely, PrP^C^ overexpression produced a permissive environment for αSynO-driven neuroinflammation. Tg20-αSynO mice exhibited increased *Tnfα* and *Il1β* expression, consistent with a possible contribution of TLR1/2-MyD88 signaling by misfolded α-synuclein described by Daniele et al. (2015). Importantly, Tg20 mice were unable to trigger the compensatory *Arginase 1* response observed in WT mice following αSynO challenge. Since *Tnfα* inhibits autophagy, promotes M1-macrophage polarization, and suppresses *Arg1* expression (Jin et al., 2018), elevated *Tnfα* levels may lock microglia into a persistent pro-inflammatory state. The selective *Arg1* upregulation observed in WT mice is particularly noteworthy. *Arg1*^+^-microglia are specialized subtype enriched in ventral striatal regions and associated with tissue repair and synaptic homeostasis (Stratoulias et al., 2023). Thus, induction of this reparative population in WT mice may represent a protective mechanism that becomes insufficient in the context of PrP^C^ overexpression. Although these bulk-tissue mRNA analyses reveal genotype- and treatment-specific neuroinflammatory transcriptional signatures, further experiments targeting protein should be conducted to confirm these findings.

Since scRNA-seq data demonstrated that *PRNP* is expressed at low levels in microglia, the inflammatory alterations observed across genotypes are unlikely to reflect direct microglial responses to PrP^C^ levels. Instead, our findings support a model in which PrP^C^-mediated neuronal stress secondarily drives microglial activation. In Tg20 mice, severe neuronal injury induced by PrP^C^-αSynO interaction may generate danger-associated signals that could promote neurotoxic microglial polarization, potentially establishing a feed-forward cycle of degeneration. In contrast, PrnpKO mice are protected from the initial neuronal insult, rendering basal inflammation insufficient to trigger dopaminergic loss. Indeed, inflammatory chemokines-mediated neuro-glial communication is known to play an important role in PD pathogenesis. Indeed, intrastriatal αSynO injection has previously been shown to induce dopaminergic degeneration associated with microglial recruitment through CCL21 signaling (Leser et al., 2025).

The morphological changes observed suggest that PrP^C^ levels may regulate microglial surveillance states and their responsiveness to pathological stimuli. Functional and biochemical studies will be needed to confirm this finding. Microglia dynamically transition from ramified surveillant states to activated phenotypes. Microglia in a surveillant state are characterized by highly ramified morphology with extensive branching, allowing continuous monitoring of the parenchyma (Nimmerjahn et al., 2005; Kettenmann et al., 2011); these changes are closely associated with functions such as phagocytosis, antigen presentation, and cytokine secretion (Ransohoff and Perry, 2009; Colonna and Butovsky, 2017). The αSynO challenge broadly increased these morphometric parameters, consistent with active phagocytic engagement. Microglia in healthy tissue display ramified morphology and actively clear misfolded protein aggregates through phagocytosis (Scholz et al., 2025). Recent evidence demonstrates that ramified microglia can efficiently engulf and degrade cellular debris and protein aggregates while maintaining their ramified morphology, with individual processes functioning as independent phagocytic compartments (Kamei and Okabe, 2023). In Tg20 mice, baseline hypertrophy and increased ramification of microglial cells suggest that chronic PrP^C^ overexpression may prime microglia toward a hypervigilant state even in the absence of pathological stimuli. This is consistent with evidence indicating that PrP^C^ participates in the regulation of microglial inflammatory responses (Shi et al., 2013; Shao et al., 2023). In parallel, autophagy-related pathways have been implicated in microglial clearance of α-synuclein aggregates and in the modulation of microglial polarization states (Choi et al., 2020; Jin et al., 2018), potentially contributing to the heightened pro-inflammatory transcriptional response observed following αSynO exposure. In contrast, PrnpKO mice displayed baseline increases in area and branching associated with elevated pro-inflammatory markers; however this activation remained uncoupled from neurodegeneration, likely due to the absence of PrP^C^-dependent neuronal injury mechanisms (Thom et al., 2022).

Some limitations should be noted. The Tg20 mice used here were maintained on a C57BL/6 background, whereas PrnpKO lines were generated on a mixed 129Sv × C57BL/6 background. This strain mismatch raises the possibility that differences attributed to PrP^C^ dosage partly reflect background/passenger-gene effects rather than a pure gene-dosage effect (Nuvolone and Aguzzi, 2015; Nuvolone et al., 2016). Moreover, for the TH immunoreactivity, cytokine, and microglial morphology analyses, a single 129Sv × C57BL/6 control group was used across both lines rather than background-matched controls for each line individually. While comparisons between vehicle and αSynO conditions were performed within each line separately for the TH and gene expression analyses, thereby limiting the influence of this design choice on those specific comparisons, the microglial morphology dataset included direct across-background comparisons, where this limitation carries greater potential for confounding. We cannot fully exclude a contribution of genetic background, independent of Prnp genotype, to the magnitude of the differences reported in these analyses. Future studies should be conducted to address these limitations. Furthermore, the vehicle-vs-αSynO comparison controls for surgery itself but does not confirm resolution of post-surgical inflammation, which would require a pre-surgical baseline not assessed here. Genotype/background comparisons within the vehicle condition may therefore reflect differences in baseline inflammatory expression or in the kinetics of post-surgical inflammation resolution rather than a pure PrPC-dosage effect, and residual surgical confounding cannot be fully excluded. In addition, TH immunoreactivity was quantified from a single 50 μm section per animal, proximal to the injection site, per our established protocol (Leser et al., 2025), rather than serial sections spanning the caudate-putamen. This limits our ability to fully capture rostro-caudal heterogeneity in TH loss. Serial sectioning in future studies will help confirm the extent of TH depletion reported here.

In conclusion, our findings suggest that PrP^C^ expression levels may be involved in nigrostriatal vulnerability to αSynO-induced neurodegeneration. We provide transcriptomic evidence that *PRNP* is significantly upregulated in idiopathic PD patients and demonstrate, through single-cell RNA-seq analysis, that this upregulation is predominantly neuronal. Our *in vivo* experiments establish a relationship between PrP^C^ levels and αSynO-induced pathology. PrP^C^ overexpression exacerbates dopaminergic vulnerability and neuroinflammatory responses, while PrP^C^ ablation confers protection. Importantly, we characterized for the first time distinct neuroinflammatory transcriptional profiles driven by PrP^C^ dosage in the context of synucleinopathy, revealing that PrnpKO mice exhibit a basal pro-inflammatory state that paradoxically protects against αSynO toxicity, whereas Tg20 mice show a permissive inflammatory environment that amplifies dopaminergic vulnerability. While PrP^C^ has been previously proposed as a potential therapeutic target in synucleinopathies based on *in vitro* studies, experimental validation *in vivo* has been lacking. Our data provide *in vivo* evidence suggesting that PrP^C^ modulation may contribute to neuroprotection in PD. By bridging human transcriptomic findings with mechanistic validation, these results support further investigation of PrP^C^ as a potential therapeutic target.

## Data Availability

The original contributions presented in the study are included in the article/Supplementary material, further inquiries can be directed to the corresponding author.
