# Supplementary material for: Cellular prion protein levels modulate nigrostriatal vulnerability to α-synuclein oligomers in Parkinson’s disease
Source: Front Neurosci. 2026 Sep 17;20:1913279. doi: 10.3389/fnins.2026.1913279 (PMC13629302; doi:10.3389/fnins.2026.1913279)
Supplement: Supplementary file 1 [file Supplementary_file_1.docx]

**Supplementary material 1** - **Methods**

**Animals**

All *in vivo* experiments were conducted following the Brazilian Experimental Animal Use Guidelines. This study was approved by the Ethics Committee of the Health Sciences Center of the Federal University of Rio de Janeiro (UFRJ) (Protocol numbers A6/19-001-16 and 025/15). The "Guide for the Care and Use of Laboratory Animals" (published by the National Academy of Sciences, National Academy Press, Washington, D.C.) was strictly followed in all experiments. All efforts were made to minimize the number of animals used and their suffering.

For the *in vivo* experiments, four mouse lines (age 8-12 weeks) were used in this work: PrP^C^ knockout animals (PrnpKO) (Büeler et al., 1992; ZrchI), PrP^C^ overexpressing mice (Tg20) (Fischer et al., 1996; Stele et al., 2006), and their respective wild-type (WT) controls (129/Sv × C57BL/6 and C57BL/6). The PrnpKO line (Büeler et al., 1992) carries a targeted replacement of codons 4–187 of the *Prnp* gene with a neomycin-resistance cassette, and was established on a mixed C57BL/6 × 129/Sv genetic background. The PrnpKO colony was maintained by PrnpKO × PrnpKO breeding, whereas the WT colony was maintained independently by WT × WT breeding; the two colonies were never intercrossed. The Tg20 line was originally generated by microinjection of a cosmid vector containing the mouse *Prnp* gene into a ZrchI PrnpKO background, resulting in mice with elevated PrP^C^ expression at approximately 8-fold normal levels from the background (Fischer et al., 1996), and then Tg20-derived colonies were subsequently backcrossed to C57BL/6, producing a predominantly C57BL/6 genetic background (Steele et al., 2006).

All mice remained with their dams until they reached 28 postnatal days (p28). After weaning, animals were divided by sex and male animals were randomly group-housed in individually ventilated cage racks at a maximum of five animals per cage. Animals were housed on pinewood shavings with ad libitum access to certified food and water, at a room temperature of 23 ± 2 °C, relative humidity of 55 ± 5%, and a 12h light/dark cycle.

**α-Synuclein oligomers (αSynO) preparation and characterization**

The preparation and characterization of αSynO were conducted following previously established protocols (Fortuna et al., 2017; Diniz et al., 2019). In brief, recombinant human α-synuclein was expressed in *Escherichia coli* (BL21(DE3) pLysS strain) utilizing the pRK172 plasmid vector. Following purification, the fractions underwent buffer exchange, quantification, and lyophilization prior to storage at −80°C. To induce oligomerization, the lyophilized protein was reconstituted in PBS and subjected to thermal incubation at 65°C for 3 to 5 days. The successful assembly of αSynO was validated by size-exclusion high-performance liquid chromatography (SEC-HPLC) and immunologically confirmed via dot blot analysis using the conformation-specific A11 antibody (1:200; Millipore EMD, Billerica, MA, USA).

***In vivo* experimental procedures**

**Stereotaxic injections**

Mice were anesthetized with acepromazine (2 mg/kg i.p.), ketamine (100 mg/kg i.p.), and xylazine (10 mg/kg i.p.) before the procedure. A midline incision was made on the scalp, and a 0.5 mm hole was drilled in the skull. For injections in the caudate putamen nuclei (*CPu*), we administered vehicle (Veh, saline), or αSynO (2 µg) (Diniz et al., 2019) using stereotaxic coordinates: -0.2 mm anteroposterior from bregma, +2.6 mm mediolateral from the midline, and 3.0 mm dorsoventral from the skull surface (-0.2AP/+2.6ML/-3.0DV). All treatments were prepared in a final volume of 2 µL and injected with a 10 µL Hamilton syringe (Hamilton, Reno, Nevada, USA) at an infusion rate of 0.1 µL/min. Mice were treated with tramadol (10 mg/kg i.p.) every 12 h for 48h after surgery. All treatments were prepared immediately before surgery and labeled with specific codes by an experimenter who did not perform the surgery (CGF and GAN). Mice were randomly allocated to experimental groups, and the researchers who performed the surgeries were blinded to the treatment group.

For each experiment, the minimum sample size for all behavior outcomes was calculated using G*Power software (Heinrich-Heine-University). In the first experiment, 10 animals were included per group (129/Sv × C57BL/6 -Veh, 129/Sv × C57BL/6 -αSynO, C57BL/6-Veh, C57BL/6-αSynO, KOPrnP-Veh, KOPrnP-αSynO, Tg20-Veh and Tg20-αSynO). In total, 83 mice were included to generate the data presented in this study. This study had a 12.6% loss rate due to anesthesia-related deaths and procedure complications, evenly distributed among the groups.

**Behavioral tests**

To assess the behavioral impairments induced by αSynO administration in mice, we conducted motor and non-motor behavioral tests between 28 and 30 dpi as described by (Leser et al., 2025).

On the first day of the behavioral test routine, animals underwent the Rotarod. The second day consisted of the Open Field Test (OFT) and Wire Hanging Test (WHT). On the last day, the Olfactory Discrimination Test (ODT) and Pole Test were performed. All motor tests were conducted before inducing the lesion to evaluate baseline motor skills of mice and ensure equivalence between groups. Non-motor tests were not performed before the surgery to prevent interference from memory and learning in subsequent test results.

All experiments were conducted after a 1-hour acclimatization period in the procedure room and during the mice's active phase, between 09:00 and 17:00, to minimize the circadian cycle's effects on animal behavior. Tests were carried out in a sound-attenuated room, illuminated with low indirect light. Prior to each test, apparatuses were cleaned with 70% ethanol, and between animals, a 10% ethanol solution was used to eliminate odor cues. The sequence of mice for each behavioral test was randomly determined, and all tests were recorded for subsequent assessment by an unbiased evaluator, who remained blinded to the treatment groups.

Since the two WT backgrounds (C57BL/6 and 129/Sv × C57BL/6) exhibited significantly different baseline performances in all motor tests, all behavioral analyses were conducted separately, comparing each transgenic line to its respective WT control.

**Rotarod test**

The Rotarod test was conducted to evaluate gross motor coordination in mice, following established procedures (Grahl et al., 2023; Nem et al., 2018). Mice were positioned on a 5 cm diameter cylindrical rotating rod, which gradually increased its rotation speed over 5 minutes from 5 to 16 rpm. Each test day included three 5-minute sessions with a 1-hour interval between them. The average latency time to fall from the rod across the three sessions was used as the outcome measure.

**Open field test (OFT)**

The Open field test was employed to assess spontaneous motor activity and anxiety-related behavior (DeFries et al., 1966). Animals were placed in the center of a square open field arena (30x30x30 cm) and allowed to explore freely for 10 minutes. The trajectory of each mouse was recorded and analyzed using *MouseGlob* software (Grahl et al., 2023; Borsoi et al., 2021), and the resulting data were subsequently exported for statistical analysis. The primary outcome for assessing general locomotor activity was the total distance traveled during the test. To examine anxiety-related behavior, we analyzed the thigmotaxic pattern in the mice's trajectories, quantifying the percentage of distance traveled within both the central (15x15 cm) and the peripheral compartments.

**Wire hanging test (WHT)**

The Wire Hanging test was used to evaluate the neuromuscular strength of mice (Grahl et al., 2023; Oliván et al., 2015). An in-house-designed transparent plexiglass box with a wire grid on its lid was utilized for this test. Mice were initially placed on top of the lid until they secured a grip on the grid. Subsequently, the lid was gently inverted, suspending the mice approximately 20 cm above a soft base to prevent potential injury in the event of a fall. The latency time for the mice to fall from the grid was measured across three attempts per day, each lasting a maximum of 5 minutes, with an intertrial interval of 1 hour. The daily average latency to fall was then calculated to assess the overall performance of the mice in the test.

**Olfactory discrimination test (ODT)**

The ODT was conducted following established procedures (Soares Rodrigues et al., 2014; Prediger et al., 2005). The test capitalizes on a mouse's natural inclination to explore areas with familiar odors. Mice with olfactory impairment tend to explore both compartments equally, indicating a lack of discrimination (Soffié and Lamberty, 1988; Prediger et al., 2005). The apparatus consists of a box (30×30×30 cm) equally divided into two connected compartments. One side is filled with clean sawdust (non-familiar compartment), while the other side contains sawdust from cages of animals isolated for 48 hours before testing (familiar compartment). During the 5-minute test, mice are placed in the middle of the olfactory discrimination box and allowed to freely explore. The tests are recorded and subsequently analyzed for the time spent investigating each compartment and the total number of crossings between sides.

**Pole test**

The pole test was utilized to assess the fine motor coordination of mice (Grahl et al., 2023; Oliván et al., 2015). In this test, each mouse was gently positioned horizontally atop a rough-surfaced vertical pole measuring 8 mm in diameter and 55 cm in height. The latency duration for the animal to adopt a vertical position facing downward (latency to turn) and the time elapsed until all four paws made contact with the ground (latency to descend) were recorded. Every experimental session comprised three tests, with a 1 h interval, and the average time was considered. When the animal was unable to turn toward the ground or fell from the pole, the recorded time was capped at 5 minutes (maximum).

**Immunohistochemistry**

Thirty days after αSynO injection, mice were anesthetized and transcardially perfused with 20 mL of phosphate-buffered saline (PBS), followed by 20 mL of 4% paraformaldehyde (PFA) for fixation. Brains were dissected, post-fixed in cold 4% PFA overnight, and serially vibratome sectioned at 50 μm. The sections were washed with PBS 3 times, permeabilized and incubated with blocking buffer (0,1M PBS, 5% normal goat serum (NGS) and 0.5% triton X-100) for 90 minutes. They were then incubated with anti-Tyrosine Hydroxylase (TH, Sigma-Aldrich, SAB4502966, 1:100), anti-Iba1, (Wako, W1W019-19741, 1:200) antibodies overnight at 4°C, then washed again with PBS and incubated with Alexa Fluor conjugated secondary antibodies (1:500) for 2 hours. The sections were counterstained with DAPI and coverslipped with fluoromount. Slices were imaged using a Leica TCS-SP5 confocal microscope. For TH intensity quantification, a single 50 μm section per animal was selected at the antero-posterior level proximal to the intrastriatal injection site, in accordance with our previously established protocol (Leser et al., 2025). Images were acquired as a Z-stack spanning the full thickness of the section, and a Maximum Intensity Projection (MIP) was generated in ImageJ (NIH) prior to quantification of TH immunoreactivity and Iba1-based microglial morphometric analysis.

**RT-qPCR**

RT-qPCR assays were performed as previously described (Lourenço et al., 2019). Briefly, total RNA was extracted from animal tissues using SV Total RNA Isolation System (Promega), following the manufacturer’s instructions. The purity and integrity of RNA preparations were checked using the 260/280 nm absorbance ratio. Only preparations with 260/280 nm absorbance ratios higher than 1.8 and no signs of RNA degradation were used. RNA concentrations were determined by absorption at 260 nm. For real-time quantitative reverse transcription PCR (qRT-PCR), 1 μg of total RNA was used for complementary DNA (cDNA) synthesis using the High Capacity cDNA Reverse Transcription kit (Thermo Fisher Scientific). Quantitative expression analysis of target genes was performed on a 7500 Fast Real-Time PCR system (Thermo Fisher Scientific) with the PowerUp SYBR Green Master Mix. β-Actin was used as an endogenous reference gene for data normalization. qRT-PCR was performed in 20 µl reaction volumes according to the manufacturer’s protocols. The inflammatory gene panel was designed to assess pro-inflammatory (*Tnfα, Il1β, Mmp9, Il6*) and anti-inflammatory *(Arg1, Tgfβ, Il10*) markers based on their established roles in PD-related neuroinflammation and microglial polarization. Primer sequences are described in Supplementary Table 1. Cycle threshold (CT) values were used to calculate fold changes in gene expression using the 2-∆∆CT*.*

**Table 1:** List of qPCR Primers used in this study

| Primer | Sequence | Amplicon Size | RefSeq Accession |
| --- | --- | --- | --- |
| Mouse β-actin | 5’-TGG ATC GGT GGC TCC ATC CTG G-3’ (fw)  5’- GCA GCT CAG TAA CAG TCC GCC TAG A-3’ (rv) | 131bp | NM_007393.5 |
| Mouse Il-1β | 5’-TAC AAG GAG AAC CAA GCA ACG A-3’ (fw)  5’-TGC CGT CTT TCA TTA CAC AGG A-3’ (rv) | 90bp | NM_008361.4 |
| Mouse Il-10 | 5’- ACC TGG TAG AAG TGA TGC CC -3’ (fw)  5’- GAA ATC GAT GAC AGC GCC TC-3’ (rv) | 120bp | NM_010548.2 |
| Mouse Tnfα | 5’-AAG TTC CCA AAT GGC CTC CC-3’ (fw)  5’- TGG TGG TTT GCT ACG ACG TG-3’ (rv) | 113bp | NM_013693.3 |
| Mouse Mmp9 | 5’- GCA GGC CGA ATA GGA GCG TC -3’ (fw)  5’- GCG TCG TGA TCC CCA CTT AC-3’ (rv) | 89bp | NM_013599.5 |
| Mouse Il6 | 5’- TAC CAC TTC ACA AGT CGG AGG C -3’ (fw)  5’- CTG CAA GTG CAT CAT CGT TGT TC -3’ (rv) | 116bp | NM_031168.2 |
| Mouse Tgfβ | 5’- TGATACGCCTGAGTGGCTGTCT-3’ (fw)  5’- CACAAGAGCAGTGAGCGCTGAA -3’ (rv) | 107bp | NM_011577.2 |
| Mouse Arg1 | 5’- CTT GCG AGA CGT AGA CCC TG -3’ (fw)  5’- TCC ATC ACC TTG CCA ATC CC-3’ (rv) | 105bp | NM_007482.3 |

**Microglia morphology analysis**

Microglia morphological parameters were analyzed using ImageJ software (v1.54; National Institutes of Health, Bethesda, MD, USA) as previously described (Heindl et al., 2018; Fernández-Arjona et al., 2017). Briefly, z-stack confocal images of Iba1 immunostained brain slices were converted to 8-bit, gray scale images. Unsharp Mask filter was applied to further improve microglial processes visualization, and Despeckle step was performed to remove background single pixel noise. Images were then binarized, and silhouette was used to calculate single cell area, perimeter, and circularity index (4π[area]/[perimeter]2) using Analyzes Particles function. Furthermore, cells were skeletonized (Skeletonize function) and analyzed using the Analyzes Skeleton plugin to quantify total, average, and maximum branch length, number of branches, number of branch ramifications (junctions), number of triple and quadruple ramification points, and number of endpoints. Finally, skeletonized microglial cells images were used to perform Sholl`s analysis using the Sholl analysis plugin, as previously described (Heindl et al., 2018; Ferreira et al., 2014) to calculate the number of intersections between cell processes and concentric circles placed between the maximum radius of the cell soma and the radius surpassing the longest branch of the cell with an 1 µm step size. For morphology quantification, 40 cells were analyzed per condition provenient from 3 animals from independent experiments. Briefly, all analyzed morphometric parameters (cell area, perimeter, circularity, sum of intersections in Sholl's analysis, total branch length, number of endpoints, number of branches, number of junctions, number of triple points, and number of quadruple points) underwent z-score standardization for normalization and Elbow method was used for determining the optimal K in K-means clustering, and identify patterns and groupings in the data.

**Bioinformatics analysis**

**Bulk transcriptomic analysis**

We initially performed transcriptomic analysis using a DNA microarray dataset obtained from postmortem substantia nigra pars compacta (SNpc) samples from patients with Parkinson’s disease (PD) and healthy controls (GSE7621; 16 PD and 9 control samples) (Lesnick et al., 2007).

Differential gene expression analysis was conducted to compare PD and control groups. The original cohort was carefully designed with PD patients and control subjects matched for age, sex, postmortem interval (PMI), and brain pH, with a mean disease duration of 12.9 ± 6.9 years in PD patients. Strict RNA quality control procedures were implemented, including RNA integrity assessment (A260/A280 ratio > 1.9 and 28S/18S ratio > 1.6), Bioanalyzer evaluation, Affymetrix microarray quality control, and exclusion of samples with poor RNA quality or prolonged agonal states. A sensitivity analysis restricted to male subjects was performed to evaluate potential sex-related effects. However, although these clinical and demographic variables were reported and controlled during cohort selection, they were not incorporated as covariates in the differential expression analyses. Additionally, no explicit correction for batch effects was reported, which represents a limitation of the original dataset.

***PRNP* multiple correlation analysis**

To investigate genes and biological processes associated with prion protein (*PRNP*, PrPc) expression in PD, we further analyzed the transcriptomic profiles of the 16 PD samples. Spearman correlation analysis was performed between *PRNP* expression and all 44,867 genes using the Hmisc package in R. Genes were considered significantly correlated with *PRNP* if they met the following criteria: Spearman correlation coefficient |r| ≥ 0.15 and p < 0.05. Gene Ontology (GO) enrichment analysis was performed using the GO Consortium (https://geneontology.org/) via the PANTHER classification system (https://pantherdb.org/), incorporating all significantly correlated genes.

To compare the *PRNP*-associated transcriptional signature between PD patients and non-neurological individuals, the same *PRNP*-centered Spearman correlation analysis was performed independently in the control samples using same correlation and significance criteria (n=9). The resulting PD and control *PRNP*-associated genes meeting the correlation criteria in both groups sets were compared to determine their overlap and the direction of their association with *PRNP*. Associations were classified as conserved when the *PRNP*-gene correlation had the same direction in PD and controls and as opposite-direction when the sign of the correlation differed between groups.

Concordance of the *PRNP*-centered correlation profiles between PD and controls was additionally assessed by calculating the Spearman correlation between the gene-wise ρ coefficients obtained in the two groups (Δρ = ρ_PD − ρ_Control). GSEA was performed for Gene Ontology Biological Process terms in R using the clusterProfiler package and human gene annotations from org.Hs.eg.db. Gene symbols were mapped to Entrez Gene identifiers prior to analysis, and GO gene sets containing between 10 and 500 genes were included. The difference in *PRNP*-gene Spearman correlation coefficients between PD and controls as the ranking metric. Enrichment significance was corrected for multiple testing using the Benjamini-Hochberg (BH) method. Positive normalized enrichment scores (NES) therefore indicate biological processes preferentially enriched among genes showing a shift toward more positive *PRNP* correlations in PD, whereas negative NES values indicate processes enriched among genes showing a shift toward more negative *PRNP* correlations in PD. Representative enrichment plots were generated using the enrichplot package, and additional graphical representations were generated using ggplot2.

**Single-cell RNA sequencing (scRNA-seq) analysis**

scRNA-seq data from human midbrain samples were obtained from the Gene Expression Omnibus (GEO; accession GSE157783). The dataset comprises single-nucleus transcriptomic profiles from postmortem midbrain tissue of 11 individuals, including six neurologically healthy controls and five patients with idiopathic PD.

Raw count matrices, gene annotations, and cell metadata were processed in R using the Seurat package. Cells expressing fewer than 200 or more than 6,000 genes, as well as cells with mitochondrial gene content greater than 10%, were excluded. Data were log-normalized, and 2,000 highly variable genes were identified using the “vst” method. Dimensionality reduction was performed using principal component analysis (PCA), followed by construction of a shared nearest neighbor (SNN) graph and clustering using the Louvain algorithm. Uniform Manifold Approximation and Projection (UMAP) was used for visualization. Cell types were annotated based on provided metadata and canonical marker genes. Gene identifiers were converted from Ensembl IDs to gene symbols using org.Hs.eg.db. Differential gene expression between IPD and control samples was performed using the Wilcoxon rank-sum test, with adjusted *p* < 0.05 considered statistically significant. Gene expression patterns were visualized using UMAP feature plots, violin plots, and dot plots.

**Statistical analysis**

In this study, we utilized different sample sizes depending on the nature of the analyses, employing 5 to 9 animals per group for behavioral assessments and 3 animals per group for molecular (RT-qPCR) and morphometric (TH immunohistochemistry) analyses. Importantly, the tissues used for molecular and morphometric evaluations were obtained from a random subsample of the exact same animals subjected to the behavioral tests. No additional animals were euthanized exclusively for these secondary analyses. The decision to process 3 samples per group rather than the entire cohort was based on practical, technical, and ethical considerations. To optimize the reliability of these data, we adopted rigorous quality control measures, including the use of validated reference genes, technical replicates, and strict RNA quality criteria for RT-qPCR. This experimental strategy maximizes the scientific yield per animal used in full compliance with responsible animal experimentation ethics.

Shapiro-Wilk and Kolmogorov-Smirnov normality tests were employed to assess normality of the data. For behavioral tests, TH immunoreactivity quantification, and microglial morphometric analyses, two-way ANOVA was performed with genotype and treatment as factors, followed by Tukey's multiple comparisons test. For RT-qPCR analysis of baseline inflammatory gene expression (vehicle-treated groups only), one-way ANOVA followed by Tukey's multiple comparisons test was used to compare the three genotypes. To assess the effect of αSynO treatment within each genotype, pairwise comparisons between vehicle and αSynO-treated animals were performed using unpaired two-tailed Student's t-test. For validation of the αSynO model in Swiss mice, unpaired two-tailed Student's t-test was used to compare vehicle and αSynO-treated groups. Bivariate correlation analyses of two continuous variables were carried out with Spearman's test. Outliers were identified and excluded as values falling outside the 99% confidence interval of the mean. All statistical analyses were performed using GraphPad Prism version 8.01 (GraphPad Prism, La Jolla, CA) or RStudio (Build 494). Statistical significance was defined as a *p*-value < 0.05 in all comparisons.

**Randomization and blinding procedures**

Animals were randomly assigned to treatment groups using a random number generator. Treatment solutions were prepared and coded by experimenters (CGF and GAN) who did not perform the stereotaxic surgeries, ensuring that surgeons remained blinded to treatment allocation throughout the procedure. Behavioral test sequences were randomized, and all behavioral tests scored by an evaluator blinded to treatment groups.

For immunohistochemical analyses, TH intensity quantification was performed using automated thresholding and particle analysis in ImageJ software with standardized parameters applied uniformly across all samples, minimizing subjective bias. Iba1^+^ microglial cell selection and imaging were performed by investigators aware of experimental groups; however, morphometric quantification was conducted using objective, pre-defined computational algorithms (ImageJ macros for area, perimeter, circularity, branch analysis, and Sholl analysis) with standardized parameters, ensuring reproducibility and reducing potential for subjective bias.

RT-qPCR sample processing, including RNA extraction, cDNA synthesis, and gene expression quantification, was performed with coded samples to maintain investigator blinding. Code assignments were revealed only after completion of data collection and primary statistical analyses for each experimental endpoint.

# **References**

Borsoi, M., Nunes, L.E.D., Barbosa, A.R., et al. (2021). Intermittent repeated stress but not ketamine changes mice response to antidepressants. Neuroscience Letters, 741, p. 135452.

Büeler, H., Fischer, M., Lang, Y., et al. (1992). Normal development and behaviour of mice lacking the neuronal cell-surface PrP protein. Nature, 356(6370), pp. 577-582.

DeFries, J.C., Hegmann, J.P. and Weir, M.W. (1966). Open-field behavior in mice: evidence for a major gene effect mediated by the visual system. Science, 154(3756), pp. 1577-1579.

Diniz, L.P., Matias, I., Araujo, A.P.B., et al. (2019). α-Synuclein oligomers enhance astrocyte-induced synapse formation through TGF-β1 signaling in a Parkinson's disease model. Journal of Neurochemistry, 150(2), pp. 138-157.

Fernández-Arjona, M. del M., Grondona, J.M., Granados-Durán, P., Fernández-Llebrez, P. and López-Ávalos, M.D. (2017) 'Microglia morphological categorization in a rat model of neuroinflammation by hierarchical cluster and principal components analysis', Frontiers in Cellular Neuroscience, 11, article 235.

Ferreira, T.A., Blackman, A.V., Oyrer, J., Jayabal, S., Chung, A.J., Watt, A.J., Sjöström, P.J. and van Meyel, D.J. (2014) 'Neuronal morphometry directly from bitmap images', Nature Methods, 11(10), pp. 982-984.

Fischer, M., Rülicke, T., Raeber, A., et al. (1996). Prion protein (PrP) with amino-proximal deletions restoring susceptibility of PrP knockout mice to scrapie. EMBO Journal, 15(6), pp. 1255-1264.

Fortuna, J.T.S., Gralle, M., Beckman, D., et al. (2017). Brain infusion of α-synuclein oligomers induces motor and non-motor Parkinson's disease-like symptoms in mice. Behavioural Brain Research, 333, pp. 150-160.

Grahl, M.V.C., Andrade, B.d.S., Perin, A.P.A., et al. (2023). Could the urease of the gut bacterium Proteus mirabilis play a role in the altered gut-brain talk associated with Parkinson's disease? Microorganisms, 11(8), p. 2042.

Heindl, S., Gesierich, B., Benakis, C., Llovera, G., Duering, M. and Liesz, A. (2018) 'Automated morphological analysis of microglia after stroke', Frontiers in Cellular Neuroscience, 12, article 106.

Leser, F.S., Júnyor, F.d.S., Pagnoncelli, I.B., et al. (2025). CCL21-CCR7 blockade prevents neuroinflammation and degeneration in Parkinson's disease models. Journal of Neuroinflammation, 22, p. 31.

Lesnick, T.G., Papapetropoulos, S., Mash, D.C., et al. (2007). A genomic pathway approach to a complex disease: axon guidance and Parkinson disease. PLoS Genetics, 3(6), e98.

Lourenco, M.V., Frozza, R.L., de Freitas, G.B., et al. (2019). Exercise-linked FNDC5/irisin rescues synaptic plasticity and memory defects in Alzheimer's models. Nature Medicine, 25(1), pp. 165-175.

Mi, H., Muruganujan, A., Ebert, D., et al. (2019). PANTHER version 14: more genomes, a new PANTHER GO-slim and improvements in enrichment analysis tools. Nucleic Acids Research, 47(D1), pp. D419-D426.

Nem de Oliveira Souza, I., Frost, P.S., França, J.V., et al. (2018). Acute and chronic neurological consequences of early-life Zika virus infection in mice. Science Translational Medicine, 10(444), p. eaar2749.

Oliván, S., Calvo, A.C., Rando, A., et al. (2015). Comparative study of behavioural tests in the SOD1G93A mouse model of amyotrophic lateral sclerosis. Experimental Animals, 64(2), pp. 147-153.

Prediger, R.D.S., Batista, L.C., Medeiros, R., et al. (2005). The risk is in the air: intranasal administration of MPTP to rats reproducing clinical features of Parkinson's disease. Experimental Neurology, 202(2), pp. 391-403.

Soares Rodrigues, L., Targa, A., Duarte Noseda, A.C., et al. (2014). Olfactory impairment in the rotenone model of Parkinson's disease is associated with bulbar dopaminergic D2 activity after REM sleep deprivation. Frontiers in Cellular Neuroscience, 8, p. 383.

Soffié, M. and Lamberty, Y. (1988). Scopolamine effects on juvenile conspecific recognition in rats: possible interaction with olfactory sensitivity. Behavioural Processes, 17(3), pp. 181-190.

Steele, A. D., Emsley, J. G., O¨zdinler, et al. (2006). Prion protein (PrP^C^) positively regulates neural precursor proliferation during developmental and adult mammalian neurogenesis. Proceedings of the National Academy of Sciences of the United States of America, 103(9), pp. 3416–3421.
